# Matched tissue-blood whole-exome sequencing improves detection of genetic etiologies in pediatric drug-resistant epilepsy

**DOI:** 10.3389/fped.2026.1751113

**Published:** 2026-02-06

**Authors:** Yuanyuan Ruan, Li Chen, Shengying Xia, Qing Lu, Jing Wang, Feng Zhu, Nannan Li, Hao Du, Dan Sun

**Affiliations:** 1 Wuhan Children’s Hospital, Tongji Medical College, Huazhong University of Science and Technology, Wuhan, China; 2 Clinic Center of Human Gene Research, Union Hospital, Tongji Medical College, Huazhong University of Science and Technology, Wuhan, China; 3 Aegicare (Shenzhen) Technology Co. Ltd., Shenzhen, China

**Keywords:** diagnostic rate, drug-resistant epilepsy (DRE), neurological development, somatic mutation, whole exome sequencing (WES)

## Abstract

**Background:**

Improving the diagnostic rate of genetic etiologies in pediatric drug-resistant epilepsy (DRE) is of critical importance, as it provides valuable guidance for clinical management in this challenging patient population.

**Methods:**

In this study, matched tissue–blood whole-exome sequencing (WES) was performed on lesional brain tissue and peripheral blood from 21 patients diagnosed with DRE who had undergone resective epilepsy surgery, in order to assess its diagnostic yield.

**Results:**

The final cohort therefore consisted of 21 pediatric patients with DRE. The patients’ ages ranged from 0.2 to 10.7 years, with a mean age of 5.2 years. Eleven were male and ten were female. Matched tissue-blood WES successfully identified the genetic etiology in six pediatric patients with drug-resistant epilepsy, yielding a diagnostic rate of 28.6% (6/21). This rate was higher than that achieved using blood-only WES (19.0%, 4/21) or clinical and imaging evaluations alone (9.5%, 2/21). Among these six positive cases: Patients 2 and 7 carried deletion and splice-site variants in the *DEPDC5* gene, respectively, and these findings were detected in both blood and diseased brain tissue; Patients 14 and 20 both had missense variants in the *TSC2* gene, detected in both blood and diseased brain tissue; Patients 8 and 16 had negative blood WES results, but somatic mosaic *BRAF* variants were detected in the diseased brain tissue, with mosaic levels of 20.2% and 13.5%, respectively.

**Conclusions:**

Matched tissue–blood WES facilitates the diagnostic yield in pediatric drug-resistant epilepsy, highlighting its critical value in detecting genetic variants that may be missed by blood-only testing and providing essential support for precision diagnosis and therapy.

## 1 Introduction

Epilepsy is a common chronic neurological disorder affecting approximately 1% of the global population, characterized by recurrent, transient disruptions of neurological function caused by abnormal neuronal discharges (1, 2). Despite the increasing availability of antiseizure medications (ASMs) in recent years, approximately 20%–40% of pediatric patients still respond poorly to existing therapies and develop drug-resistant epilepsy (DRE) (3–5). DRE is defined as the failure to achieve sustained seizure freedom after trials of two or more appropriately chosen and adequately dosed ASMs. Patients with DRE often experience frequent and difficult-to-control seizures, which significantly impair cognitive function, mental health, and quality of life, and impose a substantial burden on families and healthcare systems (6, 7).

The etiology of DRE is highly heterogeneous, encompassing structural, metabolic, immune-mediated, infectious, and genetic factors (4). Among these, genetic and developmental structural abnormalities are considered major contributors to pediatric drug-resistant focal epilepsy (4, 8). Recent studies have identified mutations in genes regulating the mechanistic target of rapamycin (mTOR) signaling pathway, including *DEPDC5*, *TSC1*, and *TSC2*, as key molecular determinants of DRE (9–11). Dysregulation of the mTOR pathway can lead to abnormal neuronal proliferation, cortical malformations, and hyperexcitability, thereby contributing to seizure refractoriness (12, 13). Advances in high-throughput genetic technologies, including whole-exome sequencing (WES) and whole-genome sequencing (WGS), have significantly improved the molecular diagnostic yield in epilepsy (8, 14). Notably, recent studies have shown that a substantial proportion of DRE patients harbor somatic mosaic mutations restricted to brain tissue (15, 16). These variants often exhibit tissue specificity and low variant allele frequency, making them difficult to detect through conventional blood-based genetic testing.

Therefore, in this study, we employed matched tissue–blood WES to identify the genetic etiologies of pediatric DRE, aiming to improve the molecular diagnostic yield and provide a foundation for elucidating its underlying genetic mechanisms and developing precision therapeutic strategies.

## 2 Materials and methods

### 2.1 Pediatric drug-resistant epilepsy cohort

All patients undergoing resection of damaged brain tissue due to DRE were recruited from Wuhan Children's Hospital. The decision to perform lesionectomy was made according to the hospital's standard clinical protocols, which involves a comprehensive assessment of seizure characteristics (at least one seizure per month with a significant impact on quality of life, meeting the criteria for DRE), disease progression, electroencephalogram (EEG) findings, and structural magnetic resonance imaging (MRI) results, in order to accurately localize the epileptogenic or suspected epileptogenic focus and network. Patient guardians’ consent and consensus from the epilepsy surgery conferences were also considered. This study included patients who underwent surgical resection for DRE between September 2021 and January 2023. Inclusion criteria were seizure onset before 14 years of age and guardian consent for comprehensive genetic testing; Patients with incomplete pathological specimens or other factors that could interfere with the assessment of brain lesions were excluded. Based on these criteria, a cohort of 21 pediatric DRE patients was established for subsequent analyses.

The study was approved by the Ethics Committee of Wuhan Children's Hospital to ensure compliance with ethical standards. Targeted WES was performed to comprehensively investigate the underlying etiologies of these patients, all of whom had previously undergone clinical and neuroimaging assessments.

Prior to participation, written informed consent was obtained from all patients or their legal guardians. The consent forms clearly explained the purpose of the study and confirmed their understanding and agreement regarding data analysis and management. The study strictly adhered to ethical and regulatory guidelines to safeguard the rights and welfare of all participants.

### 2.2 Brain tissue sampling for histopathology

Brain tissue was collected during the surgical procedure, specifically from the resected pathological regions. Upon collection, the samples were promptly fixed in formalin and subsequently embedded in paraffin. The paraffin-embedded tissues were sectioned into approximately 5 µm-thick slices, which were then stained and examined under a microscope to assess tissue architecture and cellular morphology.

### 2.3 Matched brain tissue-blood whole-exome sequencing

In this study, peripheral blood was collected from 21 pediatric patients with DRE using EDTA anticoagulant tubes, with 2 mL obtained per patient. Genomic DNA was extracted from the blood samples using the DNeasy Blood & Tissue Kit (Cat. No. 69504, QIAGEN, Germany), and WES libraries were prepared using the QIAseq Human Exome Kit (Cat. No. 333939, QIAGEN, Germany) following the manufacturer's instructions. High-throughput sequencing was performed on the Illumina HiSeq 2000 platform at a sequencing depth of 100–200×.

Resected lesional brain tissues were immediately immersed in RNAlater, incubated overnight at 4  °C, and then stored at −80  °C. Genomic DNA was subsequently extracted using the AllPrep DNA/RNA/miRNA Universal Kit (Cat. No. 80224, QIAGEN, Germany), and WES libraries were constructed with the QIAseq Human Exome Kit according to the manufacturer's protocols. High-throughput sequencing was performed on the Illumina HiSeq 2000 platform with a sequencing depth of 200–300×. The variant allele frequency (VAF) threshold was set at 1%–35% to identify somatic mosaic variants, while variants with VAF >35% were classified as heterozygous somatic mutations. To minimize false positives, analyses were performed at high sequencing depth (≥200–300×) with stringent quality control, including filtering of low-quality bases and removal of duplicate reads.

The resulting sequencing data were aligned to the hg19 reference genome for bioinformatic analyses, enabling the identification of single-nucleotide variants (SNVs), insertions, and deletions (indels), following previously described methods (17). Subsequently, the clinical significance of the detected variants was evaluated in accordance with the American College of Medical Genetics and Genomics (ACMG) guidelines (18) and the somatic variant interpretation guidelines jointly developed by AMP (Association for Molecular Pathology), ASCO (American Society of Clinical Oncology), and CAP (College of American Pathologists) (19).

### 2.4 Droplet digital PCR validation

To further verify the somatic mosaic variants identified in the lesional brain tissue, droplet digital PCR (ddPCR) was performed. An appropriate amount of frozen lesional brain tissue was used for genomic DNA extraction with the Blood/Cell/Tissue Genomic DNA Extraction Kit (centrifuge column type, YDP304, TIANGEN, China), following the manufacturer's instructions. The concentration of the extracted DNA was measured using the dsDNA HS Assay Kit (12640ES60, YEASEN, China), and DNA purity was assessed with a NanoDrop spectrophotometer. DNA samples with a concentration >5 ng/μL and an OD_260/280_ ratio between 1.6 and 2.1 were considered acceptable for subsequent analysis.

Droplet digital PCR (ddPCR) was performed using the QX200 AutoDG Droplet Digital PCR System (Bio-Rad, Hercules, CA, USA) following the manufacturer's instructions. Each 20 µL ddPCR reaction contained 10 µL ddPCR Supermix for Probes (without dUTP), 1 µL of each 10 µM primer, 1 µL of 10 µM probe, 2 µL DNA template, and 5 µL nuclease-free water. Samples were loaded into a 96-well plate, centrifuged briefly, and processed with the ddPCR Automated Droplet Generator. After droplet formation, plates were sealed and subjected to PCR amplification using the following cycling conditions: initial denaturation at 95 °C for 10 min; 40 cycles of 94 °C for 30 s (denaturation) and 55 °C for 60 s (annealing); final extension at 98 °C for 10 min; followed by storage at 4 °C. The 96-well plate was then read using the QX200™ Droplet Reader, and data were analyzed using the accompanying software to generate quantitative results. The primer and probe sequences used for ddPCR are listed in Supplementary Table 1.

## 3 Result

### 3.1 Case ascertainment and phenotypic description of the cohort

A total of 38 patients diagnosed with DRE who had undergone resective epilepsy surgery were initially included in this study. Ten patients were excluded because their parents did not provide consent for genetic testing. Preliminary assessment of the quality of resected brain tissue led to the exclusion of two additional cases that failed post-sequencing quality control. The remaining 26 patients underwent centralized evaluation of preoperative magnetic resonance imaging (MRI) and neuropathological findings. Among them, five cases were excluded according to the inclusion criteria—two due to MRI evidence of hypoxic–ischemic injury and three because the age at seizure onset exceeded 14 years.

The final cohort therefore consisted of 21 pediatric patients with DRE. The patients’ ages ranged from 0.2 to 10.7 years, with a mean age of 5.2 years. Eleven were male and ten were female. MRI revealed no detectable lesions in nine cases, while multiple abnormalities were identified in eleven cases. All patients underwent surgical resection of epileptogenic brain tissue, followed by a one-year postoperative follow-up. During this period, twelve patients (12/21, 57.1%) remained seizure-free, and nine patients (9/21, 42.9%) showed a marked reduction in seizure frequency, indicating a significant improvement after surgery (Table 1).

**Table 1:** Individuals included in the study.

Characteristics	Patients (*n*)	Percentage (%)
Age, years		
Median	5.2	
Range	0.2–10.7	
Gender		
Male	11	52.4
Female	10	47.6
MRI		
Abnormal	11	52.4
Normal	9	42.9
Surgical tissue		
Frontal lobe	6	28.6
Temporal lobe	6	28.6
Occipital lobe	3	14.3
Hippocampus	1	4.8
Prognosis after surgery		
No obvious seizures	12	57.1
Reduced	9	42.9

### 3.2 Genetic etiology diagnostic analysis

**Figure 1:**
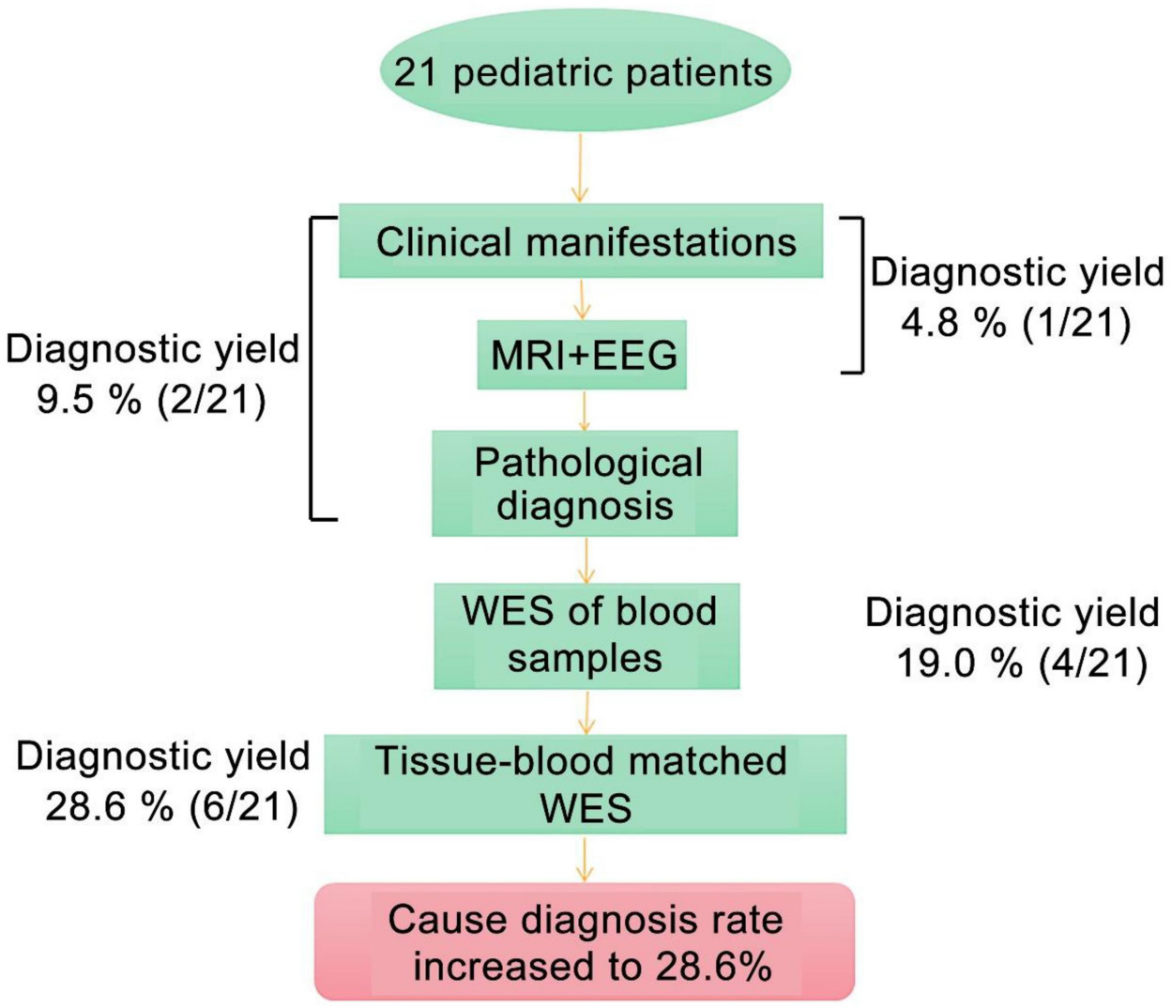
Research process diagram. MRI: magnetic resonance imaging; EEG: electroencephalography; WES: whole exome sequencing.

To investigate the genetic etiology of the 21 pediatric patients with DRE, all patients first underwent clinical evaluations, MRI, electroencephalography (EEG), and histopathological examination (Figure 1 and Supplementary Table 2). Based on these assessments, one patient was clinically diagnosed with tuberous sclerosis complex (TSC), yielding a diagnostic rate of 4.8% (1/21). Histopathological examination of resected brain tissue identified focal cortical dysplasia type IIb (FCD IIb) in patient 21, increasing the diagnostic rate to 9.5% (2/21) (Figure 1 and Supplementary Table 2). The remaining 19 patients did not receive a definitive genetic diagnosis (Supplementary Table 2). Subsequently, WES was performed on peripheral blood samples from all patients, revealing pathogenic or likely pathogenic variants in patients 2, 7, 14, and 20, involving *DEPDC5*, and *TSC2*, with a diagnostic yield of 19.0% (4/21). WES of the resected brain tissue identified pathogenic or likely pathogenic variants in patients 2, 7, 8, 14, 16, and 20, involving *DEPDC5*, *BRAF*, and *TSC2*, resulting in a markedly higher diagnostic yield of 28.6% (6/21) (Figure 1 and Table 2). The increased diagnostic rate in brain tissue WES was primarily attributable to the detection of somatic mosaic mutations in patients 8 and 16, which were not detected in peripheral blood (Table 2).

**Table 2:** Positive variants identified by WES.

ID	Blood		Brain		VT	ACMG
	Gene	Variation	Gene	Variation		
2	*DEPDC5*	NM_001242896.3:c.793_797del:p.Glu265MetfsTer9	*DEPDC5*	NM_001242896.3:c.793_797del:p.Glu265MetfsTer9	deletion	LP
7	*DEPDC5*	NM_001242896.3:c.1287 + 1G > C	*DEPDC5*	NM_001242896.3:c.1287 + 1G > C	Splicing variant	LP
8	*NPRL2*	NM_006545.5:c.1037C > T:p.Thr346Ile	*NPRL2*	NM_006545.5:c.1037C > T:p.Thr346Ile	missense variant	VUS
	*/*	/	*BRAF*	NM_001354609.2:c.1799T > A:p.Val600Glu, VAF=20.2%	mosaicism	P
14	*TSC2*	NM_000548.5:c.3598C > T:p.Arg1200Trp	*TSC2*	NM_000548.5:c.3598C > T:p.Arg1200Trp	missense variant	LP
16	*/*	/	*BRAF*	NM_001354609.2:c.1799T > A:p.Val600Glu, VAF=13.5%	mosaicism	P
20	*TSC2*	NM_000548.5:c.3532C > T:p.Gln1178Ter	*TSC2*	NM_000548.5:c.3532C > T:p.Gln1178Ter	missense variant	P

LP, likely pathogenic; P, pathogenic; VUS, variants of uncertain significance; VAF, variant allele frequency.

### 3.3 Variant and phenotype analysis

Patient 2 began experiencing unprovoked seizures at the age of 5, characterized by sudden upward eye deviation, generalized hypotonia, unresponsiveness, and limb flaccidity, with each episode lasting approximately 10 s and resolving spontaneously. Subsequently, seizure frequency gradually increased, becoming refractory to antiepileptic drugs, with up to seven episodes per day. MRI revealed no obvious abnormalities, while EEG demonstrated frequent multifocal spikes, polyspikes, spike-and-slow-wave complexes, and fast-wave rhythms, predominantly in the left anterior frontal region, with increased discharges during sleep and frequent partial seizures during wakefulness. Histopathological examination of the resected brain tissue showed clear gray-white matter demarcation, preserved cortical lamination, mildly disorganized neuronal arrangement, some neuronal atrophy or degeneration, and notable “neuronophagic” changes; gliosis was prominent without significant cellular atypia. Following surgical resection of the lesion, the patient experienced a marked reduction in seizure frequency (Supplementary Table 2). WES of both peripheral blood and the lesion tissue identified a *DEPDC5* deletion mutation (NM_001242896.3:c.793_797del:p.Glu265MetfsTer9), which was classified as likely pathogenic (LP) according to the ACMG guidelines (Table 2).

Patient 7 began experiencing unexplained seizures at the age of 2, characterized by vacant staring, limb stiffness without obvious tremor, and slowed responsiveness. The child's mental status returned to normal after each episode, and no associated symptoms such as fever, vomiting, or diarrhea were reported throughout the disease course. MRI showed no obvious abnormalities, while EEG revealed abundant spikes, sharp waves, polyspikes, spike rhythms, fast-wave rhythms, spike-and-slow-wave complexes, and irregular slow waves predominantly in the right anterior frontal region. Multiple partial seizures and frequent electrical discharges were recorded during both awake and sleep states. Histopathological examination of the resected brain tissue demonstrated preserved cortical lamination, gliosis, neuronal degeneration, and notable neuronophagic changes. Following surgical resection of the lesion, the patient remained seizure-free (Supplementary Table 2). WES of both peripheral blood and the lesion tissue identified a *DEPDC5* splice-site variant (NM_001242896.3:c.1287 + 1G > C), which was classified as LP according to the ACMG guidelines (Table 2).

**Figure 2:**
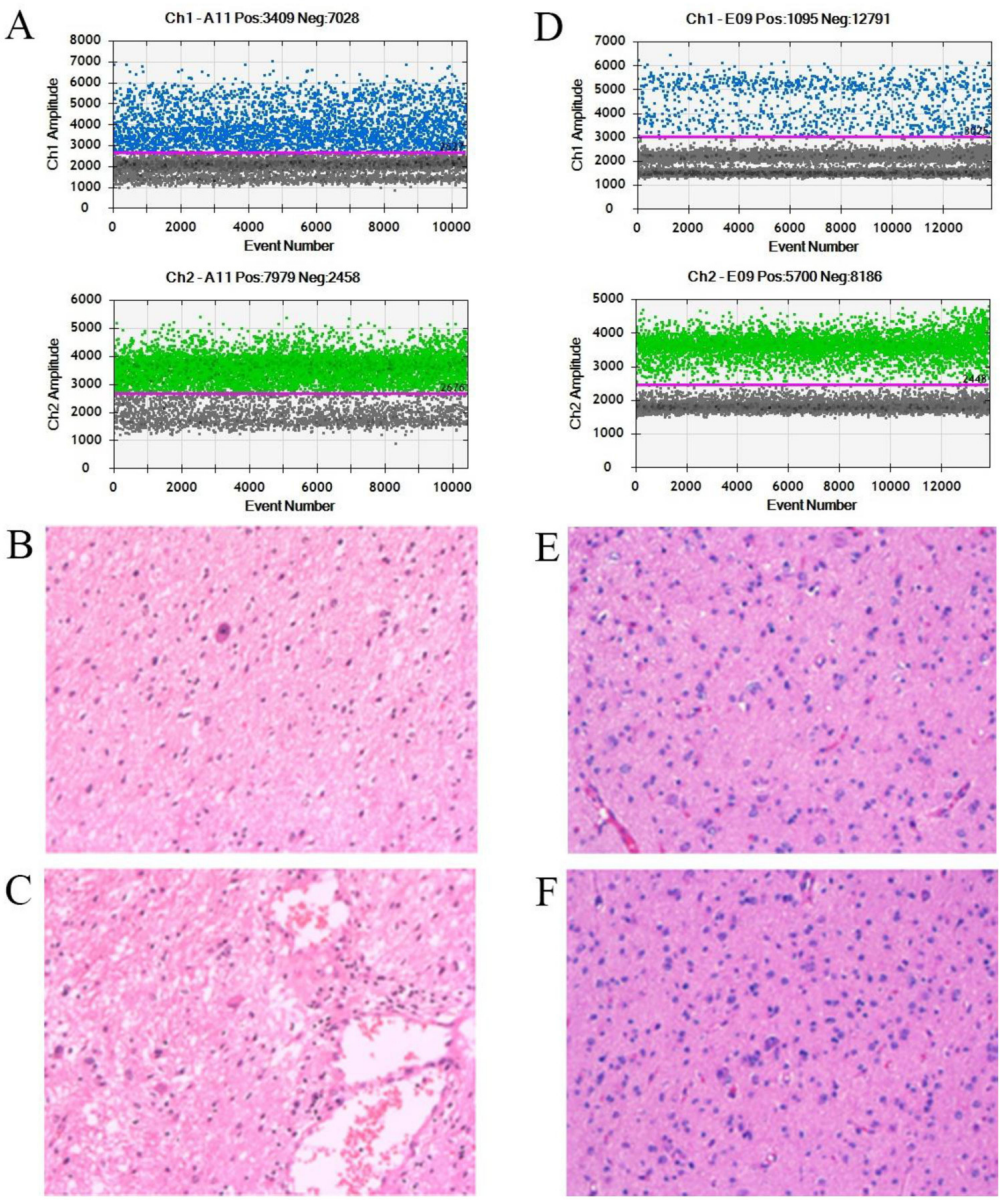
Experimental validation of somatic chimeric variants. ddPCR results and histopathological section analysis can confirm the presence of *BRAF* somatic mosaicism variants. **(A)** ddPCR results for patient 8. **(B,C)** The histopathological staining results for patient 8. **(D)** ddPCR results for patient 16. **(E,F)** The histopathological staining results for patient 16.

Patient 8 began to experience intermittent convulsions of unknown cause at the age of 5. The seizures were characterized by upward eye deviation and leftward head tilt with backward extension, without limb tremors. During the episodes, the patient was unresponsive, with each seizure lasting from several seconds to more than 10 s, and without accompanying symptoms such as fever or vomiting. Initially, the seizures occurred once every 7–8 days, but the frequency increased to once every 3–4 days as drug treatment proved ineffective. MRI examination revealed a localized softening lesion in the left occipital lobe. EEG recordings showed frequent spikes and polyspike–slow waves in the left occipital and posterior temporal regions, either sporadic or in clusters, with increased abnormal discharges during sleep and a few generalized spike and polyspike-slow waves. One partial seizure was captured during monitoring. Histopathological examination of the resected lesion revealed a mixture of atypical glial cells and neurons. The glial cells were densely arranged and enlarged in size, with microcystic changes and small foci of calcification, suggesting a possible tumor-like lesion in the left occipital lobe. Based on these features, the lesion was classified as a diffuse astrocytoma (Figures 2B,C and Supplementary Table 2). After surgical resection of the lesion, the patient's seizures were markedly improved, and no further seizures were observed during postoperative follow-up. WES of both peripheral blood and lesional brain tissue identified a missense variant in the *NPRL2* gene (NM_006545.5:c.1037C > T:p.Thr346Ile); however, according to ACMG guidelines, this variant was classified as a variant of uncertain significance (VUS). In addition, a somatic mosaic *BRAF* variant (NM_001354609.2:c.1799T > A:p.Val600Glu) was detected exclusively in the lesional brain tissue, with a VAF of 20.2% (Table 2 and Figure 2A). Based on ACMG and somatic variant interpretation guidelines, this variant was classified as pathogenic (P).

Patient 14 began experiencing epileptic seizures at two months of age with an unknown etiology. The seizures manifested as clusters of nodding, bending forward, and head-shaking movements, often accompanied by crying. Antiepileptic drug treatment showed no significant improvement. MRI examination revealed widened sulci on the surface of both cerebellar hemispheres. EEG recordings showed frequent multifocal and generalized discharges, predominantly in the left frontotemporal region, with 10 partial seizures and 2 electrographic seizures captured during monitoring. Histopathological analysis of the resected brain tissue showed poorly demarcated gray and white matter structures, localized glial proliferation, neuronal degeneration, neuronophagia, and balloon cells. Focal proliferation of glial and neuronal cells formed nodular lesions. Following surgical resection of the lesion, the frequency of seizures was markedly reduced (Supplementary Table 2). WES of both peripheral blood and lesional brain tissue identified a missense variant in the *TSC2* gene (NM_000548.5:c.3598C > T:p.Arg1200Trp), which was classified as LP according to ACMG guidelines (Table 2).

Patient 16 had experienced seizures since the age of 3 years, with no apparent precipitating factors. The seizures were characterized by brief eyelid twitching, nodding, and rapid limb jerks, often accompanied by a backward tilt of the body. These episodes occurred daily, without associated fever, vomiting, or diarrhea. Antiepileptic medications failed to achieve adequate seizure control. MRI revealed a cystic cortical lesion in the right temporal lobe. EEG recordings demonstrated frequent multifocal and generalized discharges, including spikes, polyspikes, sharp waves, spike-slow waves, and slow waves. Histopathological examination of the resected lesion confirmed a ganglioglioma in the right temporal lobe (Figures 2E,F and Supplementary Table 2). Following surgical resection, the frequency of seizures markedly decreased. WES of peripheral blood showed a negative result. In contrast, WES of the lesional brain tissue detected a somatic mosaic variant in *BRAF* (NM_001354609.2:c.1799T > A:p.Val600Glu) with a VAF of 13.5% (Table 2 and Figure 2D). According to the ACMG and the somatic variant interpretation guidelines, this variant was classified as pathogenic (P).

Patient 20 began experiencing seizures at the age of 2 years and 8 months, with no identifiable etiology. The seizures were characterized by eye blinking triggered by fear, limb stiffness or tremor, occasionally accompanied by head and eye deviation, and mild limb twitching. Each episode lasted from several tens of seconds to a few minutes and occurred frequently. MRI revealed tuberous sclerosis. EEG showed frequent generalized and multifocal discharges, most prominent in the bilateral occipital regions, which increased during sleep. Histopathological examination of the resected brain tissue demonstrated gliosis, neuronal degeneration, and the presence of neuronophagia and balloon cells (Supplementary Table 2). Following surgical resection of the lesion, the patient remained seizure-free. WES of both peripheral blood and lesional brain tissue identified a nonsense variant in *TSC2* (NM_000548.5:c.3532C > T:p.Gln1178Ter) (Table 2). According to the ACMG guidelines, this variant was classified as pathogenic.

## 4 Discussion

In this study, we performed etiological analyses in 21 pediatric patients with DRE. Clinical and imaging examinations confirmed one case of TSC and one case of FCD IIb, yielding a diagnostic rate of 9.5% (2/21). WES of peripheral blood identified four positive cases with a diagnostic rate of 19.0% (4/21), while matched blood-brain tissue WES identified six positive cases, corresponding to a diagnostic rate of 28.6% (6/21) (Figure 1 and Table 2). These findings indicate that our integrated sequencing approach improves the etiological diagnostic yield in pediatric DRE patients.

Further stratification of the identified etiologies revealed that 66.7% (4/6) of the positive cases carried pathogenic variants in genes related to the mTOR signaling pathway. Specifically, two patients harbored nonsense variants in *TSC2*, one patient carried a canonical splice-site variant in *DEPDC5*, and another had a frameshift deletion in *DEPDC5* leading to a premature stop codon. As negative regulators of the mTOR pathway, *TSC2* and *DEPDC5* mutations result in hyperactivation of mTOR signaling, thereby affecting cellular growth, differentiation, proliferation, and energy metabolism, ultimately contributing to epileptogenesis (9, 16, 20–25). Notably, patients 2 and 7 exhibited no detectable structural abnormalities on neuroimaging (Supplementary Table 2), yet harbored likely pathogenic variants in *DEPDC5* (Table 2), underscoring the critical value of genetic testing in identifying underlying causes of epilepsy.

The etiology of DRE in children is highly complex, making its treatment a long-standing challenge in clinical practice. Current therapeutic strategies mainly include ketogenic diet therapy, pharmacological treatment, vagus nerve stimulation (VNS), and cranial epilepsy surgery (6, 7, 26, 27). A study conducted in the United States compared the long-term outcomes of these approaches and showed that the 10-year survival rate was 89.27% in the medication-only group, 92.65% in the VNS combined with medication group, and 98.45% in the epilepsy surgery combined with medication group (28). These results highlight the importance of surgical intervention combined with antiepileptic drug therapy in improving the prognosis of pediatric DRE patients. In our study, all 21 patients underwent surgical resection and achieved significant improvement. During one year of follow-up, 57.1% of patients remained seizure-free, while 42.9% exhibited a marked reduction in seizure frequency (Table 1).

Although pharmacological therapy remains a key focus of clinical management, its efficacy in DRE is often limited. Notable progress has been made with molecular targeted therapies; for instance, mTOR inhibitors have demonstrated clear benefits in treating DRE associated with *TSC* mutations and show potential efficacy in cases caused by *DEPDC5* mutations (29–32). However, the clinical application of mTOR inhibitors is still constrained by factors such as a narrow therapeutic window, drug resistance, and etiological heterogeneity (31). Therefore, improving the etiological diagnostic rate and elucidating the underlying molecular mechanisms of DRE are crucial steps toward the development of precision therapies.

Additionally, somatic mosaic mutations in the *BRAF* gene were detected in the brain lesions of patients 8 and 16. These mutations could not be identified through blood WES, highlighting the advantage of matched tissue–blood WES in improving diagnostic yield. These variants may have a potential causal relationship with tumor-associated focal epilepsy. Recent studies have demonstrated that *BRAF* (NM_001354609.2:c.1799T > A:p.Val600Glu) is a key driver in the progression of astrocytomas and glioblastomas (33, 34), primarily through its effects on the MAPK signaling pathway (Ras/Raf/MEK/ERK), thereby influencing tumor cell survival and proliferation (35). Based on these studies, we speculate that the somatic BRAF variants detected exclusively in brain tissue in our study may contribute to the formation of localized epileptogenic foci in pediatric DRE by activating the MAPK signaling pathway, disrupting neuronal development, and promoting network hyperexcitability.

In summary, we highlighted the importance of improving diagnostic yield and demonstrated that matched tissue-blood WES facilitates the identification of genetic etiologies in pediatric drug-resistant epilepsy. However, our study is limited by the relatively small sample size, and the inability to capture intronic regions may have resulted in missed variants, potentially introducing some bias. Nevertheless, WES allows higher sequencing depth at lower cost, which is particularly important for detecting low-level somatic mosaicism in brain tissue.

## 5 Conclusions

In our study, matched tissue-blood WES successfully identified the genetic etiology in 6 pediatric patients with drug-resistant epilepsy, yielding a diagnostic rate of 28.6% (6/21). This rate was higher than that achieved using blood-only WES (19.0%, 4/21) or clinical and imaging evaluations alone (9.5%, 2/21). Additionally, we identified seven genetic variants associated with pediatric drug-resistant epilepsy, providing important support for subsequent analyses of pathogenic mechanisms and the development of precision medicine strategies.

## Data Availability

The original contributions presented in the study are included in the article/Supplementary Material, further inquiries can be directed to the corresponding authors.
